# Environmental interventions to reduce fear of crime: systematic review of effectiveness

**DOI:** 10.1186/2046-4053-2-30

**Published:** 2013-05-12

**Authors:** Theo Lorenc, Mark Petticrew, Margaret Whitehead, David Neary, Stephen Clayton, Kath Wright, Hilary Thomson, Steven Cummins, Amanda Sowden, Adrian Renton

**Affiliations:** 1 Department of Social and Environmental Health Research, London School of Hygiene & Tropical Medicine, 5-17 Tavistock Place, London, WC1H 9SH, UK; 2 Department of Public Health and Policy, University of Liverpool, PO Box 147, Liverpool, L69 3GB, UK; 3 Centre for Reviews and Dissemination, University of York, York, YO10 5DD, UK; 4 Social and Public Health Sciences Unit, 4 Lilybank Gardens, Glasgow, G12 8RZ, UK; 5 (formerly) School of Geography, Queen Mary, University of London, Mile End Road, London, E1 4NS, UK; 6 Institute for Health and Human Development, University of East London, Water Lane, London, E15 4LZ, UK

**Keywords:** Fear of crime, Systematic review, Built environment, Intervention effectiveness

## Abstract

**Background:**

Fear of crime is associated with negative health and wellbeing outcomes, and may mediate some impacts of the built environment on public health. A range of environmental interventions have been hypothesized to reduce the fear of crime.

**Methods:**

This review aimed to synthesize the literature on the effectiveness of interventions in the built environment to reduce the fear of crime. Systematic review methodology, following Preferred Reporting Items for Systematic Reviews and Meta-Analyses (PRISMA) guidance, was used. Studies of environmental interventions which reported a fear of crime outcome and used any prospective evaluation design (randomized controlled trial (RCT), trial or uncontrolled before-and-after study) were included. Eighteen databases were searched. The Hamilton tool was used to assess quality. A narrative synthesis of findings was undertaken.

**Results:**

A total of 47 studies were included, 22 controlled and 25 uncontrolled, with total sample sizes ranging from n = 52 to approximately n = 23,000. Thirty-six studies were conducted in the UK, ten studies in the USA and one study in the Netherlands. The quality of the evidence overall is low. There are some indications that home security improvements and non-crime-related environmental improvements may be effective for some fear of crime outcomes. There is little evidence that the following reduce fear of crime: street lighting improvements, closed-circuit television (CCTV), multi-component environmental crime prevention programs or regeneration programs.

**Conclusions:**

There is some evidence for the effectiveness of specific environmental interventions in reducing some indicators of fear of crime, but more attention to the context and possible confounders is needed in future evaluations of complex social interventions such as these.

## Background

Fear of crime is known to be associated with a number of negative mental and physical health outcomes [1]. The fear of crime may cause mental health problems directly, and it may also reduce outdoor physical activity and social interaction [1]. Conversely, mental health problems may increase fear of crime, potentially leading to a feedback effect which can exacerbate both [2,3]. Crime itself also appears to have negative impacts on health: rates of violent crime at neighborhood level have been found to be associated with a range of health behaviors and outcomes [1,4]. This suggests that interventions to reduce crime or the fear of crime may potentially be a way to improve health and wellbeing outcomes, particularly mental health, at a community level.

Furthermore, both crime and the fear of crime may be influenced by factors in the built environment. The impact of built environment factors on crime rates have been a focus of Crime Prevention Through Environmental Design (CPTED) theory, which emphasizes factors such as natural surveillance and access control as environmental determinants of crime [5,6]. Several built environment interventions are known to be effective in reducing crime, including street lighting [7] and environmental programs for robbery prevention in retail settings [8]. Fear of crime is also associated with environmental factors such as litter, graffiti and patterns of land use (for example residential versus non-residential) [1]. Combining these pathways, it appears that crime and the fear of crime may mediate the effects of the physical environment on public health [9].

Thus, interventions involving changes to the physical environment may be a promising way to address fear of crime, and the broader health and wellbeing impacts of crime. The built environment has been identified as a key locus of intervention to reduce health inequalities by addressing ‘upstream’ social determinants of health outcomes [10]. That is, environmental changes can help to address the macro-level determinants of health behaviors, not only in disadvantaged areas, but also across the population as a whole. However, this point has received less attention in the field of crime prevention [11]. (Indeed, in criminology a focus on physical environmental factors has arguably been associated with the opposite shift, away from the upstream determinants of crime and towards individual-level situational determinants.) Therefore, focusing on environmental interventions also helps to bridge the hitherto largely distinct fields of public health and crime prevention. The aim of this review was to synthesize the evidence of the effectiveness of environmental interventions on fear of crime.

## Methods

The protocol for the review is available on the National Institute for Health Research (NIHR) Public Health Research (PHR) Programme website: http://www.phr.nihr.ac.uk/funded_projects/09_3000_14.asp. The review was conducted according to Preferred Reporting Items for Systematic Reviews and Meta-Analyses (PRISMA) guidance (http://www.prisma-statement.org/).

### Search strategy and inclusion criteria

The following databases were searched: Applied Social Sciences Index and Abstracts (ASSIA), Cumulative Index to Nursing and Allied Health Literature (CINAHL), Conference Proceedings Citation Index, Criminal Justice Abstracts, Dissertation Abstracts, EconLit, Embase, Education Resources Information Center (ERIC), Health Management Information Consortium (HMIC), Inside Conferences, MEDLINE, National Criminal Justice Reference Service (NCJRS), PsycINFO, Science Citation Index, Social Policy and Practice, Social Sciences Citation Index, Sociological Abstracts, and Urban Studies Abstracts. Searches were conducted between November 2010 and January 2011. All sources were searched from inception to the most current records, without date or language restrictions. Terms used in the search included terms for fear of crime, crime, antisocial behaviour, and factors and interventions in the built environment. The full MEDLINE search strategy can be found in web-only Additional file 1 (searches for other databases used a modified form of the MEDLINE search strategy). In addition, we also searched Google and Google Scholar; scanned the reference lists of included studies; searched websites of various government bodies, research groups and other relevant organizations; and consulted the review Advisory Group.

Inclusion criteria were as follows: 1. intervention involving a substantial change to the built environment; 2. study reporting data on any fear of crime-related outcome, including perceptions or feelings of safety, estimations of one’s own risk, worry about specific crimes or crime in general, or any other crime-related affective outcome or crime-related avoidance behaviors; 3. prospective intervention evaluation of any design, including randomized controlled trials (RCTs), trials and uncontrolled before-and-after studies (with non-randomized studies required to report outcome data both before and after the intervention); and 4. study conducted in a country which is a current member of the Organization for Economic Co-operation and Development (OECD).

An initial sample of 10% of abstracts was screened by two reviewers independently and differences resolved by discussion. The remainder of the abstracts was screened by one reviewer alone. At full-text screening stage, 50% of the included studies were screened by two reviewers independently.

### Data extraction and quality assessment

Data were extracted from the studies using a standardized form, which included information on the context and setting of the study, population, methodology and findings. Data extraction and quality assessment for all studies were carried out by a single reviewer and a sample was double-checked in detail by a second reviewer.

Quality assessment for the effectiveness review was carried out using a version of the Hamilton tool [12], as modified by Thomson *et al*. [13]. This tool includes six domains: selection bias, study design, confounders, blinding, data collection, and withdrawals and dropouts. These domains were used to produce an overall quality rating: A (high quality), B (medium quality) or C (low quality), using the algorithm set out in web-only Additional file 2.

### Data synthesis

Data were synthesized narratively according to intervention type. Studies were not excluded based on their quality ratings, but more emphasis was placed on higher quality studies when reporting and interpreting the results. Meta-analysis was not carried out, owing to the substantive and methodological heterogeneity of the studies. Since many studies measure several distinct fear of crime outcomes, an indicative quantitative summary measure was developed. We standardized all measures to a 0 to 100 scale, inverted where necessary so that substantively positive findings (for example reduced fear or increased perceived safety) were expressed in the same direction (as a positive number). We then calculated a median effect size across all relevant outcomes for each study, expressing relative changes for studies using controlled designs (the difference of baseline and post-test differences between intervention and comparison group) and within-group changes for studies using uncontrolled designs (the difference between pre- and post-intervention). We also extracted data on population subgroups (age, gender, ethnicity and socioeconomic status) and conducted a separate narrative synthesis of these data to identify any equity implications.

## Results

### Flow of literature through the review

**Figure 1:**
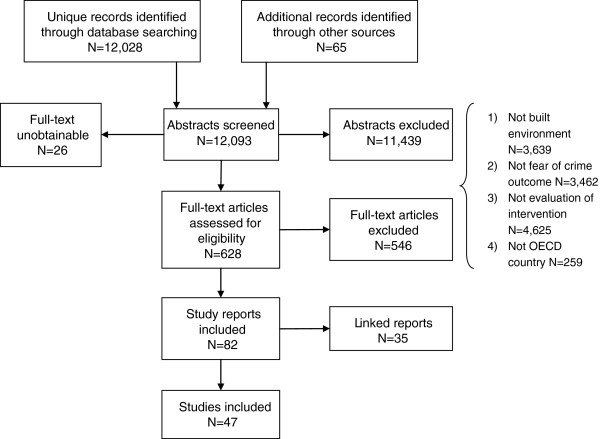
Flow of literature through the review.

Figure 1 shows the flow of literature through the review. Forty-seven studies were included in the review.

### Intervention content and quality assessment

Table 1 shows the quality ratings assigned to the studies, the study designs used and a brief description of the content of the intervention.

**Table 1:** Characteristics and quality ratings of the studies in the effectiveness review (n = 47)

**First author [reference(s)]**	**Design**	**Sample size (I/C)**	**Follow-up**	**Quality rating**	**Location**	**Intervention content**
*Category 1. Home security improvements*						
Allatt [14,15]	CBA(S)	338/322	1 year	A	Newcastle and Gateshead, UK	Improvement of residential security on deprived housing estates
Brownsell [16]	CBA(S)	24/28	5 years	A	UK, location NR	Telecare package in sheltered housing for older people
Halpern [17]	UBA(S)	55	3 years^a^	C	UK, location NR	Comprehensive renovation program on housing estate, with emphasis on security
Matthews [18]	UBA(D)	636	1 year^a^	C	Leicester, UK	Multi-component crime reduction strategy, including residential security and social components
Matthews [19]	UBA(D)	907	1 year^a^	C	Leicester, UK	Similar to Matthews [18]
*Category 2. Installation or improvement of street lighting*						
Atkins [20]	CBA(S)	248/131	2 months	A	Wandsworth, London, UK	Improvement of street lighting
Bainbridge [21]	UBA(S)	468	1 year	B	Birmingham, UK	Improvement of street lighting
Barr [22]	UBA(S)	229	2 months	C	Manchester, UK	Improvement of street lighting
Burden [23]	UBA(D)	NR	NR	C	Leeds, UK	Improvement of street lighting
Davidson [24]	UBA(S)	251	6 weeks	C	Hull, UK	Improvement of street lighting
Herbert [25,26]	UBA(S)	154	2 months	C	Cardiff, UK	Improvement of street lighting
Knight [27]	UBA(S)	125	3 weeks	C	St Helens, UK	Change of street lighting from yellow to white lights
Painter [28,29]	UBA(D)	207	6 weeks	C	North London, UK	Improvement of street lighting
Painter [29,30]	UBA(D)	143	6 weeks	C	East London, UK	Improvement of street lighting
Painter [29,31]	UBA(S)	263	13 months	B	West London, UK	Improvement of street lighting
Painter [32]	CBA(S)	431/443	1 year	A	Dudley, UK	Improvement of street lighting
Painter [33]	CBA(S)	317/88	11 months	A	Stoke-on-Trent, UK	Improvement of street lighting
Painter [34]	CBA(S)	140/167	1 year	A	Dudley, UK	Same intervention as Painter [32]
Payne [35]	UBA(S)	228	<1 year	C	Rugby, UK	Improvement of street lighting
Vamplew [36]	UBA(D)	820	<1 month	C	Middlesbrough, UK	Improvement of street lighting
Vrij [37]	UBA(D)	160	1 week	C	Enkhuizen, the Netherlands	Brighter bulbs and installation of one extra lamppost
*Category 3. Installation of closed-circuit television (CCTV) systems*						
Brown [38]	UBA(D)	699	1 year	C	Birmingham, UK	Installation of 12 CCTV cameras in city centre
Ditton [39]	CBA(D+)	1,018 total	15 months	C	Glasgow, UK	Installation of 32 CCTV cameras in city centre
Gill [40,41]	CBA(D−)	4,427/2,099	1 year	C	Several locations, England	Installation of nine different CCTV systems, four in town/city centers, five in residential areas
Musheno [42]	CBA(D+)	32/29	3 months	C	New York City, NY, USA	CCTV in public housing project with transmission to residents’ television sets
Squires [43]	UBA(D)	750	8 months	C	East London, UK	Installation of CCTV in town centre
Squires [44]	UBA(D)	243	1 year	C	Brighton, UK	Installation of 10 CCTV cameras in housing estate
*Category 4. Multi-component interventions for crime prevention*						
Arthur Young & Company [45,46]	CBA(S)	920/150	2 years^a^	A	Chicago, IL, USA	Multi-component intervention in a housing estate (locks, security personnel, entry systems, fencing, various social components)
Baker [47]	CBA(D−)	124/337	6 months	C	Pennsylvania, PA, USA	Multi-component intervention in a park (CCTV, fencing, lighting, locks, signage, cleaning, community policing, Neighborhood Watch)
Donnelly [48,49]	UBA(D)	191	5 years	C	Dayton, OH, USA	Multi-component community-based program (road closures, various social components, community-oriented policing)
Felson [50]	UBA(D)	3,581	3 years	C	New York City, NY, USA	Extensive physical redesign of bus station using CPTED principles (redesign, renovation, cleaning, lighting, information, social and policing interventions for homeless people)
Fowler [51-54]	CBA(D+)	93/798	3 years	C	Hartford, CT, USA	Multi-component community-based program (road closures, landscaping, community-oriented policing, resident organizations)
Kaplan [55]	CBA(D−)	2,772 total	6 months	C	Broward County, FL, USA	Extensive renovation work in schools using CPTED principles (renovation, reconstruction, fencing, alarms)
Kaplan [56-58]	UBA(D)	311	3 years^a^	C	Portland, OR, USA	Multi-component community-based intervention with emphasis on commercial premises (security advice, lighting, traffic calming, landscaping, cleaning, bus shelters, business organization, social programs)
Mazerolle [59,60]	RCT	Approx. 199/199	3 months	B	Oakland, CA, USA	Police-led intervention with focus on reducing disorder in the physical environment by enforcing building/housing codes
Webb [61]	CBA(D+)	Approx. 560/560	Unclear	C	London, UK	Multi-component crime prevention project on the London Underground (CCTV, passenger alarms, manned kiosks, mirrors, lighting, policing patrols)
*Category 5. Housing improvement and relocation*						
Barnes [62]	CBA(S)	199/85	6 months	C	West London, UK	Refurbishing housing association housing and relocating tenants to new, improved housing
Blackman [63,64]	UBA(S)	415	5 years^a^	C	Newcastle, UK	Housing renewal program (environmental improvements, refurbishment, demolition, security, road safety improvements)
Critchley [65]	CBA(S)	200/207	1 year	A	Liverpool, UK	Housing redevelopment with main focus on energy efficiency, security improvements (entry systems, CCTV, lighting)
Foster [66]	CBA(D−)	820/862	3 years^a^	C	East London and Hull, UK	Tenant management program including environmental improvements (security, maintenance, landscaping, entry systems)
GoWell [67,68]	CBA(D+)	6,008 total	2 years^a^	C	Glasgow, UK	Several types of regeneration and housing program, ranging from extensive rebuilding to minor renovation works
Nair [69]	UBA(S)	69	3 months	C	Glasgow, UK	Re-lighting, landscaping, housing renovation, including security improvements
Petticrew [70,71]	CBA(S)	334/389	2 years	A	Several locations, Scotland	Relocation of social housing tenants to new housing
*Category 6. Area-based regeneration initiatives*						
Beatty [72-74]	CBA(S)	19,633/4,000	6 years^a^	A	Several locations, England	Broad multi-component regeneration program, New Deal for Communities
Rhodes [75]	UBA(S)	3,459	5 years^a^	C	Several locations, England	Broad multi-component regeneration program, Single Regeneration Budget
*Category 7. Small-scale environmental improvements in public areas*						
Cohen [76]	CBA(D+)	1,535 total	3 to 14 months	C	South California, CA, USA	Improvements to public parks
Palmer [77]	UBA(D)	290	Unclear	C	Durham, UK	Bus station repainted and graffiti removed by offenders serving Community Service Orders

Sample size indicates sample at baseline in intervention and control groups, or total for non-comparative studies; ‘total’ shown for comparative studies where only total sample size is reported. Follow-up shows time from completion of intervention to latest outcome measurement for which data are available, except studies marked ‘^a^’ which indicates follow-up measured from the start of the intervention. Quality ratings: A, high quality; B, medium quality; C, low quality. CBA(S), controlled before-and-after study with same participants before and after; CBA(D+), controlled before-and-after study with different participants before and after, and with no evidence of change in population; CBA(D−), controlled before-and-after study with different participants before and after, and with some evidence of change in population; CCTV, closed-circuit television; CPTED, Crime Prevention Through Environmental Design; I/C, intervention/control group; NR, not reported; RCT, randomized controlled trial; UBA(D), uncontrolled (single-group) before-and-after study with different participants before and after; UBA(S), uncontrolled (single-group) before-and-after study with same participants before and after.

As shown in Table 1, seven categories of interventions were distinguished: 1. Home security improvements (some of these interventions also included other components); 2. installation or improvement of street lighting; 3. installation of closed-circuit television (CCTV) systems; 4. multi-component interventions for crime prevention (most focused on public areas); 5. housing improvement and relocation; 6. area-based regeneration initiatives (involving a broad range of components); and 7. small-scale environmental improvements in public areas. Of these, categories 1 to 4 include interventions whose main aim was to reduce or prevent crime and/or the fear of crime, while categories 5 to 7 include interventions which did not have such an aim, but which measured the effect of the intervention on fear-of-crime-related outcomes.

Study quality overall was generally low: 10 studies were graded as high quality (A), three as medium quality (B) and 34 as low quality (C). The generally low ratings primarily reflect two aspects of the evidence base: the large number of uncontrolled studies, or studies using an inadequately matched control group; and the incomplete reporting of methods, particularly relating to sampling and recruitment.

The findings are summarized for each intervention category. Within each category the findings from higher-quality, controlled studies are presented first, followed by those from uncontrolled studies.

### 1. Home security improvements

Five studies were identified in this category, all from the UK. The interventions covered a range of approaches. One study was narrowly focused on providing security improvements to homes [14], while the other studies combined home security with a range of other security and non-security improvements. In one study, security improvements were undertaken within the context of a telecare intervention for older people in sheltered housing [16]. In another study, the security improvements formed part of a broader program of work, which also included broader improvements to homes as well as to the surrounding area (including improved lighting, landscaping and alley gating) [17].

Two controlled studies found reductions in fear of crime as a result of home security improvements (15% [14] and 16% [16]). One uncontrolled study reported similar reductions (18% [17]), while two other uncontrolled studies found no change in fear of crime outcomes (0.1% across both studies [18,19]).

Overall, the evidence indicates that home security improvements in a range of different contexts may be promising for reducing fear of crime.

### 2. Installation or improvement of street lighting

Sixteen studies, four controlled and twelve uncontrolled, investigated the effects of street lighting improvements on fear of crime. Fifteen studies were conducted in the UK and one in the Netherlands.

Fourteen studies looked at the effect of improving lighting at an area level on fear of crime in general. Four of these studies used controlled designs [20,32-34]. Of these, two found that the intervention did not reduce fear (−1.5% for one study [33]; effect sizes were not clearly reported in the other [20]). One study found mixed results depending on the exact fear variable used, with significant improvements in two of five analyses (aggregate effect size 2.7% [32]). The fourth study found a significant improvement in fear in a sample of young people (9.8% [34]).

Ten further studies used uncontrolled designs to investigate similar interventions. Most of these studies found some improvement, although significance was often not reported. Seven studies showed a trend towards reduced fear (5.6% [21], 6% [25], 8% [23], 8% [24], 17% [30], 22% [28] and 35% [31]), and three studies showed no change or a trend towards increased fear (0% [36], −1% [22] and −6% [35]).

Finally, two studies investigated smaller-scale changes, both using uncontrolled designs. One study investigated a change from conventional yellow sodium lighting to a new bulb type with a whiter light in North West England [27], and the other study investigated an increase in brightness, carried out in Enkhuizen, the Netherlands [37]. Both studies found significant reductions in fear of crime (19.7% [27] and 15.1% [37]).

Overall, the evidence regarding lighting is rather mixed. While the uncontrolled studies showed reductions in fear, these were generally not replicated in more rigorous studies, although some of the latter studies did show some positive effects.

### 3. Installation of closed-circuit television (CCTV) systems

Six studies investigating the effect of CCTV on fear of crime were identified, five studies from the UK and one study from the USA. Three controlled studies were identified. One of these studies was incompletely reported and did not appear to show any substantial change in fear levels [39]. The second controlled study, carried out in New York City, NY, USA, investigated the effect of the installation of CCTV in a public housing project with transmission to residents’ television sets. It showed very mixed findings, with improvements in some fear outcomes and adverse changes in others (median 9.8% [42]). The third and largest study concerned the effects of installations of CCTV in four town centers and five residential areas in England. It found some within-group reductions in fear (median within-group effect, 5%), but little evidence of reduced fear when the control group was taken into account: out of sixteen analyses reported, only six showed positive comparative trends in fear outcomes (of which two were statistically significant) [40].

Three uncontrolled studies in England evaluated the impact of CCTV. Two studies showed slight positive trends (2% [38] and 2.5% [43]), and one study showed a negative trend (−7.5% [44]). Statistical significance was not measured in any of these studies.

Overall, the evidence tends to show that CCTV is not effective in reducing fear of crime, although the quality of the evidence is limited.

### 4. Multi-component interventions for crime prevention

Nine studies investigated large-scale programs of environmental change to address crime or the fear of crime. Eight studies were based in various cities in the USA and one study was based in London, UK. Intervention components often included security improvements, lighting improvements and installation of CCTV, as well as more general environmental improvements such as landscaping or graffiti removal. In addition, many included non-built-environment components such as changes to policing practice (for example community-oriented policing) and/or social programs (for example drug treatment, employment initiatives). A range of settings were investigated, including residential and business areas, parks, schools and public transport stations.

Three controlled studies reported trends towards reductions in fear of crime [45,51,61]. Two of these studies investigated broad environmental crime reduction programs in residential areas in Chicago, IL, USA (6% [45]), and Hartford, CT, USA (4% [51]), which in one case was reported to reach significance, although the analysis is non-standard [51]. The third study focused on public transport stations in London and showed a positive trend in fear, although significance was not reported (7.5% [61]).

However, two further controlled studies showed small and non-significant adverse trends towards increased fear: one study of a police-led intervention in Oakland, CA, USA, to address environmental problems at crime ‘hot spots’ (change scores not reported [59]), and the other study of a security program in schools in Broward County, FL, USA (−3.8% [55]).

Two uncontrolled studies also showed reductions in fear of crime: one study of a police-led intervention in a crime ‘hot spot’ in a park in Pennsylvania, PA, USA (29.8% [47])^a^, and the other study in a public transport setting in New York City (21% [50]), although significance was not reported. One further uncontrolled study of an intervention focusing on road closures in Dayton, OH, USA, showed a non-significant trend toward reduced fear (6% [48]). A fourth uncontrolled study showed mixed results, although with a median positive trend (5% [56]).

Overall, the findings on multi-component environmental crime reduction programs are mixed and do not constitute strong evidence of effectiveness in reducing fear.

### 5. Housing improvement and relocation

Seven studies, all carried out in UK cities, examined the effects of housing improvement on fear of crime outcomes. Three studies included renovation of existing housing [63,66,69], one study focused on the provision of new housing and relocation of residents [70], and three studies included elements of both [62,65,67].

Three controlled studies all showed small and non-significant improvements in fear of crime (2.5% in Liverpool [65] and 7% in London [62]; change scores are not reported for the third study, carried out in Hull and London [66]). Four uncontrolled studies showed varying results, with two studies showing significant reductions in fear of crime (9.1% [70]^b^ and 16.1% [63]), one study showing no change (1.5% [69]), and one study showing significant adverse effects in fear (−19% [67]).

The findings on housing are very mixed. The variation in findings does not seem to depend on whether studies involved substantial relocation or not (as might be hypothesized, since relocation would disrupt social networks, thus potentially increasing fear of crime). However, there are some positive findings from reasonably robust studies.

### 6. Area-based regeneration initiatives

There were two studies in this category, involving large-scale nationwide urban regeneration programs conducted in the UK. Both the Single Regeneration Budget (SRB) [75] and the New Deal for Communities (NDC) [72] included a wide range of component initiatives (for example housing, environmental improvement, employment and economic development initiatives, and crime prevention). In both studies there was no clear trend in fear outcomes. The SRB evaluation showed a 2% within-group improvement [75]. The NDC evaluation showed substantial improvements in the intervention group over the timeframe of the evaluation (median within-group change, 13.5%), but similar improvements were also found in the matched comparison group, so may not be attributable to the intervention (median comparative change, −2.5%) [72].

### 7. Small-scale environmental improvements in public areas

One controlled study from the USA and one uncontrolled study from the UK looked at small-scale environmental improvements which were not primarily aimed at reducing crime. In the USA study, new gym equipment was installed in urban parks in Southern California, and general environmental improvements were carried out [76]. The UK study was set in Durham in the North of England, and involved cleaning and repainting a bus station [77]. Both studies found significant improvements in at least some fear of crime outcomes (change scores not reported in one study [76]; a significant 8.1% improvement in perceived risk, but no significant change in feelings of safety (change scores not reported) in the other study [77]).

### Population subgroups and inequalities

Most information on the differential effect of interventions related to age and gender. Ethnicity was investigated in one study alone [72] and the effects of socioeconomic status, or relevant proxy measures, on outcomes were not investigated in any studies. Supplementary Additional file 3 discusses the findings and presents harvest plots for age, gender and ethnicity.

The findings on age were mixed, with some studies showing greater effect in older people and some showing greater effect in younger people. The findings on gender tended to show slightly greater improvements for women than for men. However, the only study to directly measure an interaction between intervention exposure and gender was the non-crime-focused intervention in Durham, which found consistently greater effectiveness for men than women across four outcomes; however, only in one outcome did the difference reach significance [77].

### Discussion and conclusions

The findings of this review indicate that some environmental interventions may have the potential to reduce fear of crime, although in no case is the evidence conclusive. The most promising categories of intervention appear to be home security improvements (category 1), at least in certain contexts, and general environmental improvements, for which some limited evidence was found (category 7). In most other categories there was little evidence of reducing fear of crime. Where positive findings emerged from non-comparative studies, they were rarely confirmed by more robust designs with matched comparison groups. In some cases, such as street lighting (category 2), effect sizes found in controlled studies were lower than in uncontrolled studies.

Installation of CCTV (category 3) appeared to be the least promising of the interventions, with consistent evidence of ineffectiveness in reducing fear of crime; however, it is possible that these findings may be confounded if CCTV was installed in areas known to be crime ‘hot spots’.

Findings in the other four categories were more equivocal, with some positive findings, though none provide strong evidence of reductions in fear of crime. Of concern is the finding from one study of increased fear of crime [67]. This was a large housing improvement program in Glasgow, Scotland; the study authors speculate that relocation may have had disruptive effects on social networks, hence increasing fear of crime. This hypothesis remains to be tested.

In general, the evidence base covered in the review has considerable limitations. Study quality is generally poor, with very few studies using robust designs with adequately matched control groups, and many studies not conducting appropriate statistical analyses. For those categories containing few rigorously conducted studies, CCTV and multi-component environmental programs in particular, the results should be regarded as indicative only.

One of the most serious limitations of the evidence base is the heterogeneity in the outcome measures aggregated as ‘fear of crime’ in this review. This heterogeneity, and the associated problems with the concept of fear of crime itself, have been subject to sustained critique. The different definitions of fear have been shown to lead to widely varying empirical results and arguably do not access the same underlying construct [78-81]. Supplementary Additional file 4 shows the different types of fear of crime outcome measures included in the studies; as shown, there is wide variation in the types of outcome included. While we have simply aggregated these distinct outcomes in this review as though they referred to the same construct, it should be borne in mind that the heterogeneity of measures places limitations on what can be inferred from the results. In particular, several of the studies with the most positive trends only measured perceived safety, rather than affective fear; [50,61,76,77] such outcomes could be hypothesized to have a lesser impact on wellbeing.

Positive mental health is increasingly recognized as an important aspect of public health and may be influenced by good living environments, housing, employment, transport, education and a supportive political structure; and at a community level, by a sense of belonging, social support, a sense of citizenship and participation in society [82]. Fear of crime is one important mechanism (or pathway) which mediates the relationship between these influences and mental health and wellbeing. This review has found some support for the role of physical environment as a target for effective interventions, although further evaluative evidence is needed. As both crime and mental health and wellbeing are strongly socially patterned, future research should also have a clearer equity focus particularly taking account of socioeconomic status, ethnicity and gender.

## Endnotes

^a^ The Pennsylvania, PA, USA, study [47] was conceived as a controlled design, but is treated in this review as uncontrolled because the comparison group appear to have benefited from the intervention as much as the ‘intervention’ group.

^b^ The Petticrew *et al*. study in Scotland [70] used a controlled design, but only within-group findings are available for fear outcomes.

## Abbreviations

ASSIA: Applied Social Sciences Index and Abstracts; CCTV: closed-circuit television; CINAHL: Cumulative Index to Nursing and Allied Health Literature; CPTED: crime prevention through environmental design; ERIC: Education Resources Information Center; HMIC: Health Management Information Consortium; NCJRS: National Criminal Justice Reference Service; NDC: New Deal for Communities; NIHR: National Institute for Health Research; OECD: Organization for Economic Co-operation and Development; PHR: Public Health Research; PRISMA: Preferred Reporting Items for Systematic Reviews and Meta-Analyses; RCT: randomized controlled trial; SRB: Single Regeneration Budget

## Competing interests

The authors declare that they have no competing interests.

## Authors’ contributions

The idea for the study was formulated by MP and MW, with input from HT, SCu, AS and AR. Searches were conducted by KW. Screening and coding were conducted by TL, DN and SCl. TL conducted the data analysis and wrote the first draft of the manuscript. All authors contributed to revising the manuscript and approved the final draft.

## Supplementary Material

Additional file 1 MEDLINE search strategy. Click here for file

Additional file 2 Quality assessment for the systematic review of effectiveness. Click here for file

Additional file 3 Findings on population subgroups. Click here for file

Additional file 4 Fear of crime outcome measures. Click here for file
