# Supplementary material for: Environmental interventions to reduce fear of crime: systematic review of effectiveness
Source: Syst Rev. 2013 May 12;2:30. doi: 10.1186/2046-4053-2-30 (PMC3660218; doi:10.1186/2046-4053-2-30)
Supplement: Additional file 1 — MEDLINE search strategy. [file 2046-4053-2-30-S1.doc]

**Additional file 1. MEDLINE search strategy**

1 (fear$ adj3 crime$).ti,ab. (104)
2 (worry$ adj3 crime$).ti,ab. (0)
3 (worries adj3 crime$).ti,ab. (0)
4 (worried adj3 crime$).ti,ab. (0)
5 (anxiety adj3 crime$).ti,ab. (11)
6 (anxious adj3 crime$).ti,ab. (0)
7 perceived safety.ti,ab. (153)
8 (perception$ adj3 safety).ti,ab. (411)
9 (insecurit$ adj3 crime$).ti,ab. (3)
10 (feeling adj (safe or unsafe)).ti,ab. (98)
11 fear/ or stress, psychological/ (86736)
12 crime/ (11244)
13 11 and 12 (237)
14 1 or 2 or 3 or 4 or 5 or 6 or 7 or 8 or 9 or 10 or 13 (903)
15 crime/ (11244)
16 rape/ or sex offenses/ or homicide/ (18596)
17 (rape or murder or homicide).ti,ab. (9456)
18 (sexual adj (assault or violence)).ti,ab. (2786)
19 antisocial behavior$.ti,ab. (1682)
20 antisocial behaviour$.ti,ab. (439)
21 anti social behavior$.ti,ab. (46)
22 anti social behaviour$.ti,ab. (61)
23 public disorder.ti,ab. (9)
24 social disorder.ti,ab. (60)
25 (disorderly adj2 behaviour$).ti,ab. (3)
26 (disorderly adj2 behavior$).ti,ab. (4)
27 graffiti.ti,ab. (67)
28 vandal$.ti,ab. (150)
29 delinquent behaviour$.ti,ab. (119)
30 delinquent behavior$.ti,ab. (532)
31 (noise adj (pollution or nuisance)).ti,ab. (197)
32 nuisance neighbor$.ti,ab. (0)
33 nuisance neighbour$.ti,ab. (0)
34 (incivility or incivilities).ti,ab. (62)
35 hooligan$.ti,ab. (25)
36 mugging.ti,ab. (14)
37 verbal abuse.ti,ab. (355)
38 rowdiness.ti,ab. (1)
39 street litter$.ti,ab. (2)
40 (flyposting or fly posting or flytipping or fly tipping).ti,ab. (1)
41 15 or 16 or 17 or 18 or 19 or 20 or 21 or 22 or 23 or 24 or 25 or 26 or 27 or 28 or 29 or 30 or 31 or 32 or 33 or 34 or 35 or 36 or 37 or 38 or 39 or 40 (36776)
42 urban space$.ti,ab. (44)
43 urban design$.ti,ab. (66)
44 urban plan$.ti,ab. (287)
45 urban ecology.ti,ab. (33)
46 urban geography.ti,ab. (7)
47 urban renewal.ti,ab. (43)
48 urban regeneration.ti,ab. (23)
49 city space$.ti,ab. (2)
50 city design$.ti,ab. (65)
51 city plan$.ti,ab. (97)
52 city ecology.ti,ab. (0)
53 city geography.ti,ab. (0)
54 town space$.ti,ab. (0)
55 town design$.ti,ab. (8)
56 town plan$.ti,ab. (89)
57 town ecology.ti,ab. (0)
58 town geography.ti,ab. (0)
59 (public space or public spaces).ti,ab. (175)
60 (neighborhood$ or neigbourhood$).ti,ab. (8661)
61 (street or streets).ti,ab. (7075)
62 built environment.ti,ab. (462)
63 housing estate$.ti,ab. (142)
64 (problem estate$ or sink estate$).ti,ab. (1)
65 public housing.ti,ab. or public housing/ (1019)
66 ((council or social) adj housing).ti,ab. (158)
67 (housing adj3 (improv$ or initiative$)).ti,ab. (326)
68 (housing adj2 design$).ti,ab. (100)
69 housing project$.ti,ab. (149)
70 ((renovat$ or repair$) adj3 (home or homes or house or houses or housing)).ti,ab. (152)
71 (park or parks or carpark$).ti,ab. (8090)
72 (campus or campuses).ti,ab. (3640)
73 "Transportation"/ (4978)
74 public transport$.ti,ab. (532)
75 railway$.ti,ab. (1142)
76 (bus or buses).ti,ab. (1795)
77 Environment Design/ (2472)
78 Urban Renewal/ (637)
79 greenways.ti,ab. (11)
80 (landscape adj (plan$ or design$)).ti,ab. (59)
81 physical environment.ti,ab. (1510)
82 (civic adj (design$ or space$ or plan$)).ti,ab. (1)
83 residential area$.ti,ab. (1424)
84 residence characteristics/ (13987)
85 (city adj (centre or centres or center or centers)).ti,ab. (454)
86 (shopping adj (centre or centres or center or centers or mall or malls)).ti,ab. (271)
87 (area based adj2 (initiative$ or intervention$)).ti,ab. (18)
88 42 or 43 or 44 or 45 or 46 or 47 or 48 or 49 or 50 or 51 or 52 or 53 or 54 or 55 or 56 or 57 or 58 or 59 or 60 or 61 or 62 or 63 or 64 or 65 or 66 or 67 or 68 or 69 or 70 or 71 or 72 or 73 or 74 or 75 or 76 or 77 or 78 or 79 or 80 or 81 or 82 or 83 or 84 or 85 or 86 or 87 (54820)
89 streetlight$.ti,ab. (18)
90 street light$.ti,ab. (36)
91 alley gat$.ti,ab. (1)
92 alleygat$.ti,ab. (0)
93 (neighborhood watch or neighbourhood watch).ti,ab. (4)
94 (cctv or closed circuit television or video surveillance).ti,ab. (546)
95 security camera$.ti,ab. (6)
96 broken window$.ti,ab. (19)
97 hot spot polic$.ti,ab. (1)
98 gated communit$.ti,ab. (1)
99 defensible space$.ti,ab. (3)
100 designing out crime.ti,ab. (0)
101 CPTED.ti,ab. (3)
102 target hardening.ti,ab. (1)
103 89 or 90 or 91 or 92 or 93 or 94 or 95 or 96 or 97 or 98 or 99 or 100 or 101 or 102 (637)
104 14 or 41 (37387)
105 88 or 103 (55387)
106 104 and 105 (1194)
