# Supplementary material for: Environmental interventions to reduce fear of crime: systematic review of effectiveness
Source: Syst Rev. 2013 May 12;2:30. doi: 10.1186/2046-4053-2-30 (PMC3660218; doi:10.1186/2046-4053-2-30)
Supplement: Additional file 2 — Quality assessment for the systematic review of effectiveness. [file 2046-4053-2-30-S2.doc]

**Additional file 2. Quality assessment for the systematic review of effectiveness**

*Methods*

The quality assessment tool contains six questions:
1. Selection bias
2. Study design
3. Confounders
4. Blinding
5. Data collection
6. Withdrawals and dropouts.

Each question can get an A (high), B (medium) or C (low) quality rating, as per the tool below. The overall rating for the study is then calculated on the following basis:

A = A for q2 and A/B on at least two of qq1,3,6;
B = A for q2 and A/B on one of qq1,3,6; or B for q2 and A/B on at least two of qq1,3,6;
C = A for q2 and C for all of qq1,3,6; or B for q2 and A/B on less than two of qq1,3,6; or C for q2.

The guidelines for the specific questions are as follows.

1. Selection bias

| Selected study sample very likely to represent population from target area AND 80 to 100% response at baseline | A |
| --- | --- |
| Selected study sample very likely to represent population from target area AND 60 to 79% response at baseline; OR  Selected study sample somewhat likely to represent population from target area AND 80 to 100% response at baseline | B |
| <60% baseline response; OR  Somewhat likely to represent population AND <80% response; OR  Not likely to represent population OR representativeness NR/unclear; OR  Response rate at baseline NR/unclear | C |

1. Study design

| Control group and pre and post longitudinal data OR random allocation | A |
| --- | --- |
| No control group and pre and post longitudinal data; OR  Control group and pre and post cross-sectional data AND no indication of  major change in population | B |
| No control group and pre and post cross-sectional data; OR  Control group and pre and post cross-sectional data AND possibility of  major change in population | C |

Note: ‘longitudinal’ = same individuals pre and post; ‘cross-sectional’ = different individuals. Where studies use mixed designs (e.g. presenting both cross-sectional and longitudinal data), give the highest grade applicable to the analyses actually reported. Where studies collect longitudinal data and report attrition rates, grade as longitudinal even if only cross-sectional analyses are reported.

1. Confounders

| Control group matched on key variables (at least two of: crime rate (area level), SES or relevant proxies (area or individual level), gender, age, ethnicity (individual level)) AND supporting data presented; OR  Outcomes adjusted for key variables (at least two of: gender, age, ethnicity, SES) using appropriate methods | A |
| --- | --- |
| Stated that control group matched or ‘similar’, but supporting data not presented | B |
| No matching or adjustment reported AND likely to be substantial differences between groups; OR no information on differences between intvn and control group; OR no control group | C |

Note: RCTs will be graded ‘B’ if no information on between-group differences is presented

1. Blinding

| Both outcome assessors AND participants blind to allocation | A |
| --- | --- |
| Either outcome assessors OR participants blind to allocation | B |
| Blinding NR; OR no control group | C |

1. Data collection

| Piloting or pre-testing of tool; OR checks on validity of data (e.g. verification of a percentage of responses); OR tool shown to be reliable in relevant population | A |
| --- | --- |
| Data collection tool based on previous research, but no piloting or checking, and reliability not demonstrated | B |
| Data collection unclear; OR tools not piloted, checked or based on previous research | C |

1. Withdrawals and dropouts

| Attrition <20% | A |
| --- | --- |
| Attrition 21%-40% | B |
| Attrition >40%; OR attrition NR; OR cross-sectional data only | C |

Note: Attrition is measured as the percentage of the baseline sample lost at final follow-up

*Results*

The results of quality assessment are shown in Table 1.

Table 1. Results of quality assessment for the effectiveness studies (N=47)

| Study code | Design | 1. Selection bias | 2. Study design | 3. Confounders | 4. Blinding | 5. Data collection | 6.Withdrawals | OVERALL |
| --- | --- | --- | --- | --- | --- | --- | --- | --- |
| Category (1). Home security improvements | | | | | | | | |
| Allatt | CBA(S) | A | A | B | C | C | B | A |
| Brownsell | CBA(S) | C | A | B | C | C | B | A |
| Halpern | UBA(S) | C | B | C | C | C | C | C |
| Matthews a | UBA(D) | C | C | C | C | A | C | C |
| Matthews b | UBA(D) | C | C | C | C | A | C | C |
| Category (2). Street lighting | | | | | | | | |
| Atkins | CBA(S) | C | A | A | C | A | B | A |
| Bainbridge | UBA(S) | B | B | C | C | B | B | B |
| Barr | UBA(S) | C | B | C | C | B | C | C |
| Burden | UBA(D) | C | C | C | C | C | C | C |
| Davidson | UBA(S) | C | B | C | C | B | B | C |
| Herbert | UBA(S) | C | B | C | C | B | A | C |
| Knight | UBA(S) | C | B | C | C | B | C | C |
| Painter a | UBA(D) | C | C | C | C | A | C | C |
| Painter b | UBA(D) | C | C | C | C | B | C | C |
| Painter c | UBA(S) | A | B | C | C | B | B | B |
| Painter d | CBA(S) | B | A | A | B | A | A | A |
| Painter e | CBA(S) | A | A | A | B | B | A | A |
| Painter f | CBA(S) | B | A | A | B | C | B | A |
| Payne | UBA(S) | C | C | C | C | B | C | C |
| Vamplew | UBA(D) | B | C | C | C | C | C | C |
| Vrij | UBA(D) | C | C | C | C | C | C | C |
| Category (3). CCTV | | | | | | | | |
| Brown | UBA(D) | C | C | C | C | C | C | C |
| Ditton | CBA(D+) | A | B | C | C | C | C | C |
| Gill | CBA(D−) | B | C | B | C | C | C | C |
| Musheno | CBA(D+) | C | B | B | C | B | C | C |
| Squires a | UBA(D) | C | C | C | C | B | C | C |
| Squires b | UBA(D) | C | C | C | C | C | C | C |
| Category (4). Multi-component crime prevention | | | | | | | | |
| Arthur Young & Co. | CBA(S) | C | A | B | C | A | B | A |
| Baker | CBA(D−) | B | C | C | B | B | C | C |
| Donnelly | UBA(D) | C | C | C | C | C | C | C |
| Felson | UBA(D) | B | C | C | C | C | C | C |
| Fowler | CBA(D+) | B | B | C | C | A | C | C |
| Kaplan a | CBA(D−) | C | C | C | C | C | C | C |
| Kaplan b | UBA(D) | C | C | C | C | C | C | C |
| Mazerolle | RCT | C | A | A | B | A | C | B |
| Webb | CBA(D+) | C | B | C | C | C | C | C |
| Category (5). Housing improvement | | | | | | | | |
| Barnes | CBA(S) | C | A | C | C | A | C | C |
| Blackman | UBA(S) | B | B | C | C | B | C | C |
| Critchley | CBA(S) | C | A | B | C | B | B | A |
| Foster | CBA(D−) | B | C | A | C | B | C | C |
| GCPH | CBA(D+) | C | B | A | C | A | C | C |
| Nair | UBA(S) | B | B | C | C | C | C | C |
| Petticrew | CBA(S) | C | A | A | C | A | B | A |
| Category (6). Regeneration | | | | | | | | |
| Beatty | CBA(S) | B | A | B | C | A | C | A |
| Rhodes | UBA(S) | B | B | C | C | C | C | C |
| Category (7). Other environmental interventions (non-crime-focused) | | | | | | | | |
| Cohen | CBA(D+) | C | B | A | C | C | C | C |
| Palmer | UBA(D) | C | C | C | C | B | C | C |
