# Supplementary material for: Environmental interventions to reduce fear of crime: systematic review of effectiveness
Source: Syst Rev. 2013 May 12;2:30. doi: 10.1186/2046-4053-2-30 (PMC3660218; doi:10.1186/2046-4053-2-30)
Supplement: Additional file 3 — Findings on population subgroups. [file 2046-4053-2-30-S3.doc]

**Additional file 3. Findings on population subgroups**

This appendix briefly describes the findings on subgroups, with associated harvest plots. Each bar represents a study, with shorter bars representing lower-quality studies, and longer higher-quality; the placing of the bar represents the median difference in effect sizes between the relevant groups. The harvest plots show only those studies which present fully differentiated data for relevant subgroups. Studies which focused on particular populations but did not discuss differential effects between subgroups, and studies which presented incomplete information about subgroup effectiveness, are briefly mentioned in the text but are not included in the harvest plots. In few cases were subgroup analyses tested for significance, and all but one (Allatt) concern only within-group findings, so these results should be treated with caution.

*Age*

Findings on age are mixed. Of the three studies which present full subgroup data and are shown in the first harvest plot in Figure 3.1, two show little difference between older people (over 60 or 65) and the rest of the population (Herbert; Beatty), while one shows a very substantially greater effect in older people than in the whole population (Allatt). However, in the comparison between younger people (under 25 or 35) with the whole population (the second harvest plot), one study shows greater effectiveness in young people (Beatty), and one less (Herbert).

Of the studies which present partial data on age subgroups, and are not included in the harvest plots, three show no clear difference (Arthur Young & Co.; Atkins; Bainbridge), and one finds adverse effects (increased fear) for older people but not other age groups (Kaplan b).

Of those studies targeted at particular age groups, both the two aimed at older people (Brownsell; Painter c) and the one aimed at children and young people (Painter f) find substantial positive effects in fear outcomes.

**Figure 3.1. Harvest plots for age**

*Gender*

The findings for gender, represented in the harvest plot in Figure 3.2, tend to show a slightly greater effect of interventions on women than on men, although the differences in many cases are relatively small. It should also be noted that the only study (Palmer) to directly measure an interaction between intervention exposure and gender, and test it for significance, finds consistently greater effectiveness for men than women, and that this difference reached significance for one of four outcomes.

Of studies which present partial data on gender and are not included in Figure (Arthur Young & Co.; Atkins; Bainbridge; Gill), none show a substantial difference between men and women. The study by Halpern finds substantial positive effects on both fear of crime outcomes and mental health status outcomes for a sample of women (Halpern).

**Figure 3.2. Harvest plot for gender**

*Ethnicity*

Only one study compared outcomes by ethnicity. Beatty et al. (Beatty) find slightly greater improvements in fear and perceived safety outcomes in non-White than White participants.

**Figure 3.3. Harvest plot for ethnicity**
