# Supplementary material for: Environmental interventions to reduce fear of crime: systematic review of effectiveness
Source: Syst Rev. 2013 May 12;2:30. doi: 10.1186/2046-4053-2-30 (PMC3660218; doi:10.1186/2046-4053-2-30)
Supplement: Additional file 4 — Fear of crime outcome measures. [file 2046-4053-2-30-S4.doc]

**Additional file 4. Fear of crime outcome measures**

Table 4.1 sets out the types of outcome measures included under ‘fear of crime’ in the review. (Only measures for which complete pre- and post-test outcome data are reported are included here; however, index measures which are only reported as aggregates are represented by their components.) These outcome measures include:

- Affective measures of fear (e.g. How afraid / concerned / worried are you ...?) about crime in general;
- Affective measures of fear (e.g. How afraid / concerned / worried are you ...?) about specific types of crime;
- Cognitive measures of perceived risk, safety or likelihood of victimisation about crime in general;
- Cognitive measures of perceived risk, safety or likelihood of victimisation about specific types of crime;
- The effects of fear (e.g. How much is your quality of life affected by fear of crime?);
- Avoidance behaviours (e.g. avoiding certain areas), either explicitly due to fear of crime or more generally;
- Feelings of safety (e.g. How safe do you feel?) or unsafety; or
- Perceptions of the fear or risk experienced by others (e.g. How much is fear of crime a problem in this area?; How risky is it for women to go out alone after dark?).

As Table 4.1 shows, the effectiveness studies bear out researchers’ concerns about the heterogeneity of outcome measures used to investigate fear of crime. The most commonly used types of measure are feelings of safety (N=31) and worry about specific crimes (N=19), but no single type of measure is universally used. Moreover, it should be noted that there is considerable heterogeneity even within the eight subcategories identified in Table 4.1. For example, this analysis does not disaggregate: fear or feelings of safety with respect to different times of day (day/night), or to different places, which are measured in several studies; time-specific (frequency) versus non-time-specific measures; nor the various vocabularies used for affective measures (‘fear’, ‘worry’, ‘concern’ etc.). All of these apparently subtle distinctions may have substantial impacts on research findings.

**Table 1. Fear of crime outcome measures reported in the intervention studies (N=47)**

| Reference | Fear / worry / concern, general | Fear / worry / concern about specific crimes | Perc safety / risk, general | Perc safety / risk of specific crimes | Effects of fear (e.g. on quality of life) | Avoidance behaviours | Feelings of (un)safety | Perc of others’ fear/risk |
| --- | --- | --- | --- | --- | --- | --- | --- | --- |
| Category (1). Home security improvements | | | | | | | | |
| Allatt | ✓ | ✓ |  |  | ✓ |  |  |  |
| Brownsell | ✓ |  |  |  |  |  | ✓ |  |
| Halpern |  | ✓ |  |  |  |  |  |  |
| Matthews a |  | ✓ |  |  |  | ✓ |  |  |
| Matthews b |  | ✓ |  |  |  |  |  |  |
| Category (2). Street lighting | | | | | | | | |
| Atkins |  | ✓ |  | ✓ |  |  | ✓ |  |
| Bainbridge | ✓ | ✓ |  |  |  |  | ✓ | ✓ |
| Barr |  | ✓ |  |  |  | ✓ | ✓ | ✓ |
| Burden |  | ✓ |  |  |  | ✓ |  |  |
| Davidson |  | ✓ |  |  |  | ✓ | ✓ | ✓ |
| Herbert |  | ✓ |  |  |  | ✓ | ✓ | ✓ |
| Knight |  |  |  |  |  |  | ✓ |  |
| Painter a |  | ✓ |  |  |  |  |  |  |
| Painter b |  | ✓ |  |  |  |  | ✓ |  |
| Painter c |  | ✓ |  |  |  | ✓ |  | ✓ |
| Painter d |  |  | ✓ |  |  | ✓ | ✓ | ✓ |
| Painter e | ✓ | ✓ | ✓ |  |  | ✓ | ✓ | ✓ |
| Painter f |  | ✓ | ✓ |  |  | ✓ | ✓ | ✓ |
| Payne |  | ✓ |  |  | ✓ |  |  |  |
| Vamplew | ✓ |  |  |  |  | ✓ | ✓ |  |
| Vrij |  |  | ✓ |  |  |  | ✓ |  |
| Category (3). CCTV | | | | | | | | |
| Brown |  |  |  |  |  |  | ✓ |  |
| Ditton | ✓ |  |  |  |  | ✓ | ✓ |  |
| Gill | ✓ |  |  |  |  | ✓ | ✓ |  |
| Musheno |  |  |  |  |  | ✓ | ✓ |  |
| Squires a |  |  |  |  |  |  | ✓ |  |
| Squires b |  |  |  |  |  |  | ✓ |  |
| Category (4). Multi-component environmental crime prevention | | | | | | | | |
| Arthur Young & Co. | ✓ |  |  |  |  |  |  |  |
| Baker |  |  |  |  |  |  | ✓ |  |
| Donnelly |  |  | ✓ |  |  |  |  |  |
| Felson |  |  | ✓ |  |  |  |  |  |
| Fowler |  | ✓ |  | ✓ |  |  |  |  |
| Kaplan a |  | ✓ |  | ✓ |  |  |  |  |
| Kaplan b |  | ✓ |  | ✓ |  | ✓ | ✓ | ✓ |
| Mazerolle |  |  |  |  |  | ✓ | ✓ |  |
| Webb |  |  | ✓ |  |  |  |  |  |
| Category (5). Housing improvement | | | | | | | | |
| Barnes |  |  |  |  | ✓ |  | ✓ |  |
| Blackman |  |  | ✓ |  |  |  | ✓ |  |
| Critchley |  |  |  |  |  |  | ✓ |  |
| Foster |  | ✓ |  |  |  |  | ✓ |  |
| GCPH |  |  |  |  |  |  | ✓ |  |
| Nair |  | ✓ |  |  |  | ✓ | ✓ | ✓ |
| Petticrew |  |  | ✓ |  |  |  | ✓ |  |
| Category (6). Regeneration | | | | | | | | |
| Beatty |  | ✓ |  |  |  |  | ✓ |  |
| Rhodes |  |  | ✓ |  |  |  |  |  |
| Category (7). Other environmental interventions (non-crime-focused) | | | | | | | | |
| Cohen |  |  | ✓ |  |  |  |  |  |
| Palmer |  |  |  | ✓ |  |  | ~ |  |
